# First line in psychiatric emergency: pre-hospital emergency protocol for mental disorders in Iran

**DOI:** 10.1186/s12873-020-00313-2

**Published:** 2020-03-16

**Authors:** Fatemeh Shirzad, Fatemeh Hadi, Seyede Salehe Mortazavi, Maryam Biglari, Hassan Noori Sari, Zeinab Mohammadi, Mehrdad Kazemzade Atoofi, Seyed Vahid Shariat

**Affiliations:** 1 Spiritual Health Research Center, Iran University of Medical Sciences, Tehran, Iran; 2 Mental Health Research Center, Department of Psychiatry, School of Medicine, Iran University of Medical Sciences, Tehran, Iran; 3 School of Behavioral Sciences and Mental Health (Tehran Institute of Psychiatry), Iran University of Medical Sciences, Tehran, Iran; 4 Preventive Medicine and Public Health Research Center, Iran University of Medical Sciences, Tehran, Iran; 5 Deputy of Technical and Operations of the Emergency Organization, Tehran, Iran; 6 Emergency Organization, Tehran, Iran; 7 Mental Health Research Center, School of Behavioral Sciences and Mental Health (Tehran Institute of Psychiatry), Tehran, Iran

**Keywords:** Mental disorder, Protocol, Emergency, Pre-hospital

## Abstract

**Introduction:**

This article is a report of designing a rapid and effective guide for paramedics who take care of patients in a pre-hospital setting to answer developing demands.

**Methods:**

The relevant literature was reviewed, and the topics were extracted. Then, the extracted items were discussed in an expert panel. Finally, items were discussed in a meeting including emergency technicians and emergency technical assistants to identify implementation problems.

**Results:**

Important topics for managing psychiatric patients were categorized at three levels: 1) Patient safety and security issues, 2) Patient status assessment and diagnosis, and 3) Patient management (medical, behavioral management, and referral to a treatment center).

**Discussion:**

This protocol can be a solution to improve emergency technician training. Such summarized protocols can be used for rapid review immediately before exposing a patient with an acute psychiatric condition. Due to specific cultural and different access to medicines in Iran, some issues are different.

## Background

Mental health problems are quite prevalent and pose a heavy burden on a society. According to the Burden of Disease study performed in Iran in 2003, mental and behavioral disorders were ranked as the second in intentional and unintentional injuries [1]. Moreover, the latest national survey on mental disorders has shown that a one-year prevalence of psychiatric disorders in Iran is 23.6% [2]. A small but significant proportion of these patients would need intensive care, sometime during the course of their disease. In such a psychiatric emergency, paramedics are in the front line of emergency medical service (EMS) for assessing, managing and transferring patients to the emergency wards of hospitals. Therefore, the role of paramedics is critical in rapid and accurate decision making.

Despite the vital role of paramedics in pre-hospital management of psychiatric problems, little evidence exists on the issue [3]. Paramedics have three major needs for a good performance: knowledge, skill for an appropriate clinical decision making, and organizational factors [3]. Educational courses have been shown to improve their knowledge, attitude, skill, and self-efficacy in performing the job and also will help them in better decision making on the scene [4–6].

Emergency paramedic may face many difficulties in making decisions for emergency patients with mental illness [7]. They should prioritize safety in the first place and simultaneously assess the patients for possible physiological, psychiatric, pathological, socio-cultural, and legal aspects to reach an accurate diagnosis and also manage plans [8, 9]. Additionally, they are frequently exposed to the aggressive behavior of violent patients [10] and inappropriate management of aggressive behavior can have severe consequences. In the case of highly aggressive patients, chemical and physical restraint should be performed very cautiously [11] under clinical guidelines [10].

Emergency staffs are at the risk of cumulative stress because of the high amount of stress they experience; and if it is not adequately addressed, emotional trauma and its related dysfunction would appear [12]. Paramedic’s workloads for mental illness are growing and their ability for decision making in the complex situation of psychiatric emergency should be amended [4]. Furthermore, their ability to triage patients with psychiatric problems is limited and, in some cases, paramedics admitted the necessity of assessment tools and training courses for maintaining the capability of emergency services [4, 6, 12, 13]. Therefore, they will benefit from educational courses and rapid clinical protocols that would help them with their clinical decision making [6]. This article is a report of the process of designing a rapid and effective guide for paramedics who take care of patients to answer increasing demands in a pre-hospital setting.

## Methods

This project was conducted at the request of the Technical Assistance and Operations Department of the Iranian Emergency Organization. Firstly, the relevant literature was reviewed. To prevent bias and to enrich resources, in addition to the chapters on managing patients with acute psychiatric symptoms in psychiatric textbooks, more specialized resources in the field of emergency psychiatric patient’s management were also reviewed. We also searched for recent related articles in PubMed, Scopus, and Web of Knowledge databases.

We combined keywords related to emergency situation including “psychiatric emergency”, “behavioral emergency”, “agitated behavior”, “agitation”, “violence”, and “aggression” with terms of “management”, “protocol” and “prehospital care”. Mesh terms, expert opinion, and some keywords from the existing literature review were used to select these keywords. Then, we screened the retrieved articles and removed the unrelated articles.

17 documents out of 56 primitive cases were selected which their information has been presented in Table 1. The criterion for omitting the articles was decided by research team based on the critical appraisal using peer review checklist for protocol recommended by Cochrane Community [14].

**Table 1:** The main data of the Included studies

Title	Author/s	Time	Design	Target population	objective	Main points/ result	Sample size
Emergency psychiatry: physical and chemical restraint in the psychiatric emergency service	Currier GW., et al	2000	Review	Patient with psychiatric emergency symptoms	Comparison of benefit and risk of physical restrain in aggressive patient. Comparison of chemical and physical restrain in aggressive patient. Provide recommendations about The correct method of physical restrain	Physical and chemical restraints are important for optimal management of agitated and aggressive patient	–
Pharmacological management of acute agitation	Battaglia J.	2005	Clinical experience	Patient with agitation	Comparison of different medications for Rapid tranquillization of agitated patients in emergencies	The use of ziprasidone (IM) and olanzapine is effective in controlling agitation in patients.	–
A growing evidence base for management guidelines: Revisiting Guidelines for the Management of Acutely Disturbed Psychiatric Patients	Macpherson R., et al	2005	Review	Patient with aggression	Providing recommendations and appropriate drugs to psychiatric management patients with aggression	Four levels of observation of patients at risk of violence were founded. Appropriate medications for rapid tranquilization have been obtained. Appropriate recommendations for aggressive management patients included adjustment of environment and verbal relaxation	–
The expert consensus guideline series. Treatment of behavioral emergencies	Allen M., et al	2005	Expert panel	Psychiatric patient with acute symptoms	Identifying and defining important factors for formulating recommendations and guidelines for agitation control in emergency patients	Defining the following elements: the threshold for emergency interventions, the scope of assessment for varying levels of urgency and cooperation, guiding principles in selecting interventions, and appropriate physical and medication strategies at different levels of diagnostic confidence and for a variety of etiologies and complicating conditions.	50 experts
Early psychological intervention in prehospital emergency care systems.	Cunha S., et al	2009	RCT	Patient with agitation	assessment of the effect of antipsychotics in agitation control of psychiatric emergency patients	Olanzapine and haloperidol are the best and safe drugs to control patients’ agitation	–
Atypical antipsychotic medications in the management of disruptive behaviors in children: safety guidelines and recommendations	McKinney C., et al	2011	Review	Children and adolescents with Disruptive Behavior	providing guidelines for the use of antipsychotics in children	Studies do not prove the safety of prescribing antipsychotics in children. Further and more detailed studies are needed.	–
Haloperidol for psychosis-induced aggression or agitation (rapid tranquillization). Cochrane database of systematic reviews	Powney MJ., et al	2012	Systematic review	Psychiatric patient with agitation	Assessment effect of haloperidol (oral, IM and IV) in agitated patient	Haloperidol can be the first choice in controlling the restless of psychiatric patients and can be quite redemptive in their treatment	32 studeis
The psychopharmacology of agitation: consensus statement of the American Association for Emergency Psychiatry Project BETA Psychopharmacology Workgroup.	Wilson MP., et al	2012	Review	Patient with agitation	providing a specific guideline for the management of agitation in different situations	The main treatment for agitation should be based on the most likely cause	–
Issues in the management of acute agitation: how much current guidelines consider safety? Frontiers in psychiatry	Pacciardi B	2013	Review of guideline	Patient with acute agitation	assessment of available and safe drugs for rapid tranquilization in agitative Patient in Emergency	The use of second-generation of antipsychotic is preferred	11 guidlines
Intravenous droperidol or olanzapine as an adjunct to midazolam for the acutely agitated patient: a multicenter, randomized, double-blind, placebo-controlled clinical trial	Chan EW., et al	2013	RCT	Patient with agitation	to determine the efficacy and safety of intravenous droperidol or olanzapine as an adjunct to intravenous midazolam for rapid patient sedation.	Intravenous droperidol or olanzapine as an adjunct to midazolam is effective and decreases the time to adequate sedation compared with midazolam alone.	336 patient
Risk for physical restraint or seclusion in the psychiatric emergency service (PES). General hospital psychiatry	Simpson SA., et al	2014	Retrospectively reviewed medical records, nursing logs, and quality assurance data for all adult patient encounters in a PES Over a 12-month period	Adult patient encountered in a PES	describe risk factors associated with patients experiencing physical restraint or seclusion in the psychiatric emergency service (PES)	Physical restraint when needed can be very helpful in calming agitated and violent patients	5333 patiant
Assessment and management of agitation in psychiatry: expert consensus. The world journal of biological psychiatry.	Garriga M., et al	2016	Systematic review and Delphi and expert panel	Patient with agitation	provide comprehensive recommendations for agitation evaluation and management	physical restrain without over sedation is useful for agitation management. Underline medical illness should be considered.	124 papers
Haloperidol plus promethazine for psychosis-induced aggression. Cochrane Database of Systematic Reviews.	Huf G., et al	2016	Systematic review	Psychotic patient with aggression	assessment The safety and effect of the combination of promethazine and haloperidol vs haloperidol alone in an agitated patient	This combination is effective and safe but haloperidol alone is better	6 studies
Biological treatment of acute agitation or aggression with schizophrenia or bipolar disorder in the inpatient setting. Annals of clinical psychiatry	Correll CU., et al	2017	Review	Patient with agitation	summarize the available biological treatment options for patients with schizophrenia or bipolar disorder presenting with acute agitation or aggression in the inpatient setting	The use of second-generation of antipsychotic is preferred. The use of drugs should be based on patient tolerated	–
Managing acutely aggressive or agitated people in a psychiatric setting: a survey in Lebanon.	Dib JE., et al	2018	A survey of clinicians’ opinions and practice was conducted between July and August 2017 at the largest psychiatric hospital in Lebanon	Psychiatric Patient with acute symptoms	Surveying treatments in a psychiatric setting in Lebanon	There was a consistent view that there should first be verbal control, then use of medications, and finally physical restrain of the patient It was found evidence of high family involvement in psychiatric emergency	seven experienced psychiatrists
Managing agitation associated with schizophrenia and bipolar disorder in the emergency setting.	Zeller SL., et al	2015	Review of (BETA) guidelines with the addition of data on new pharmacologic interventions	Patient with agitation	Raise awareness of best practices for the management of agitation in the ED and to consider the role of new pharmacologic interventions in this setting.	(BETA) guidelines address the complete management of agitation Newer modes of delivery that could be useful in rapidly managing agitation include inhaled, buccal/sublingual and intranasal formulations Non-invasive formulations have the potential to improve overall patient experience	–
Pharmacologic Management of Agitation and Aggression in a Pediatric Emergency Department–A Retrospective Cohort Study.	Kendrick JG., et al	2018	Retrospective observational study	Patients less than 20 years of age with agitation and aggretion	To describe medication utilization in the management of agitation and aggression in a pediatric ED and to assess the safety of their use.	Benzodiazepine and antipsychotic drug therapy for acute agitation and aggression in children appears to be safe and well tolerated	120 patient

Finally, we extracted the topics in the remaining related articles. The extracted topics were then classified based on seven criteria as follows: 1) Order of execution (at what stage they should execute), 2) first encounter with the patient and maintaining safety, 3) Evaluation and taking a medical history, 4) The primary differential diagnoses, 5) Behavioral and drug therapy and management, 6) Decreasing life-threatening risks in patients, and 7) Repeatable rate in different sources. Based on these categories, an early draft of the protocol was written. The draft was sent to 10 psychiatrists who expert in emergency psychiatry and their initial feedbacks were collected. Some changes were made in the protocol, according to the experts’ comments. In the next step, an expert panel was held.

The panel included experts from psychiatry, forensic medicine, clinical psychology, emergency medicine, Emergency Technical Assistant (deputy) and a general physician. In the panel, each expert commented on each of the items and stages according to the following questions:

1. To what extent is it necessary?
2. To what extent is it clear to the emergency technician?
3. To what extent is it generalizable to similar symptoms in other emergencies (e.g., acute restlessness in non-psychiatric patients)?
4. To what extent can it be done according to the equipment and situation of our society?
5. To what extent has patient safety been considered at this stage?
6. To what extent has the safety of emergency technicians been considered?
7. To what extent is the safety of the patient’s relatives and those present at the scene taken into account?
8. Considering the current law, how legal is any of the actions of emergency technicians?

Each item was scored using a Likert scale from very low [1] to very high [9]. After adding the scores based on the expert’s decisions, Items with a mean score 6 and higher, remained unchanged in the protocol and those with a mean score of 3 and lower were eliminated from the protocol. Other Items were discussed once more and were finally agreed upon with some minor changes.

The above case was presented in a meeting of 60 emergency technicians (with more than 15 years of working experience) from different cities of Iran. Applicability, clearness and comprehensibility of items were discussed. The ambiguities of the protocol were addressed, and the implementation issues of the protocol were identified. The protocol was finalized in 2 pages that were applicable in an ambulance for reviewing important points when confronting with psychiatric patients.

## Results

Based on the findings from the literature review and discussed issues in the expert panel, important topics for managing psychiatric patients were categorized in three levels: 1) Patient safety and security issues, 2) Patient status assessment and diagnosis and 3) Patient management (medical, behavioral management, and referral to a treatment center).

### Primary actions

The first step is to ensure the safety of the patient, technicians, and people on the scene [15]. This stage includes: a) pre-scene assessments of site security, escape routes, and safe locations in the event of violence from the patient, b) assessment of patient’s access to weapons and equipment that could threaten his/her own life, technicians or attendees [16], C) Assessment for risk and need for back up and the presence of police, which includes anticipating their entrance method and avoidance of entering the place alone, D) using family capacities to provide security [16] and E) Assessment of risk factors for violence and predicting it Symptoms of imminent aggression include: 1) Motor restlessness and agitation, 2) The loud and threatening tone of voice, 3) Threatening behavior and gestures, 4) Verbal Threats, 5) Staring and angry face mode, 6) Sudden behaviors (He throws the object in his hand suddenly), 7) Bizarre behavior due to delusion and hallucination.

### Patient assessment

Patient evaluation includes the following: A) Urgent physical needs by evaluating vital signs (Airway, presence of respiratory distress, and pulse), B) Obtaining a targeted mental health history of the patient from his or her family including: Demographic characteristics (sex, age, occupation), history of psychiatric illness, history of physical and primarily neurological diseases, history of drug abuse, history of violence or suicide [15], C) Differential diagnoses (psychological causes versus physical causes of symptoms) and physical risk factors (sudden onset of symptoms without previous history, age younger than 12 years and older than 60 years, known neurological diseases such as seizures or dementia, existence of neurological symptoms such as ataxia, nystagmus and complex drug regimen (Table 2) [17] D) Considering cultural and spiritual aspects of patients which can effect on symptoms and how to help them.

**Table 2:** Variety of physical origin of psychiatric emergency

Intoxication/withdrawal	Medical status
• Alcohol intoxication/ withdrawal/delirium tremens • Substance intoxication/ withdrawal: - Opioids - Amphetamines - cannabis - others • Medical intoxication/ withdrawal: - Benzodiazepines - Anticonvulsants - others	• Head trauma • Post-ictal condition • Delirium • Hypoglycemia • Electrolyte disturbance (Hyponatremia, hypernatremia, hypokalemia, hyperkalemia …) • Hypoxia • Encephalitis, Meningitis • Encephalopathy (due to medical condition) • Environmental toxicity • Thyroid dysfunction

### Patient management

Patient management includes: a) behavioral management, b) pharmacological management, c) patient family management.

- Patients’ behavioral management includes following recommendations on how to behave and speak to the patient:

- Speak to the patient in a calm, measured and confident tone
- Reduce external stimuli, such as the noise and the provocative behavior of others [18]
- Reduce internal triggers like hunger and thirst, and offer water and food to the patient whenever possible.
- have empathic and non-judgmental attitudes and behaviors
- accept the patient’s hallucinations and delusions appropriately
- Don’t make a false promise to the patient
- Use short, simple sentences and repeat the sentences if necessary
- Listen to the patient
- Use patients’ words as much as possible
- Reassure the patient that you understand the problem
- Encourage the patient to provide information to those who can help
- Attempt to meet the patient’s spiritual needs (include general spiritual principles in the patient-therapist relationship, showing compassion and unconditional acceptance to the patient)
- Encourage the patient to provide information to those who can assist him/her.

In case of aggression, in addition to the mentioned points, Keep the patient at least 2 m away, tell the patient that aggression is unacceptable, offer medication and in order to prevent harm themselves or others, use physical restraint if s/he continues [19].

b) Pharmacological management: There are several important principles to consider in drug administration. The aim of emergency medical treatment should be to calm down the agitated patient as quickly as possible without reducing the patient’s level of consciousness. Like all emergencies, oral drugs are preferred to injectable ones. The drug should be selected based on the onset of action and availability. Short-acting drugs are preferred over long-acting drugs. Medicines with fewer side effects are also preferred. Thus, in the first step, oral medications such as benzodiazepines or lorazepam with or without typical antipsychotics such as risperidone may be used.

In the second line, other antipsychotics such as haloperidol may be administered. If the patient’s condition does not improve or he/she does not cooperate in the treatment, intramuscular antipsychotics such as Amp haloperidol 5 mg along can be used. If necessary, these medications can be repeated with cardiac and blood pressure monitoring. Other medicines such as promethazine or injectable benzodiazepines may also be used to increase the effectiveness of the administered drugs [19–21] (Table 3).

**Table 3:** Medical management

First line	Second line
1. Tab Risperidone 2 mg 2. ± Tab lorazepam 2 mg 3. Tab olanzapine 5–10 mg	Tab Haloperidol 5 mg ± Tab Lorazepam
Amp Haloperidol 5 mg ± amp lorazepam	Amp Promethazine 50 mg IM

Restraints may use with the pharmacological methods. This treatment option is used as the last choice in patients who are uncooperative and physically dangerous and may harm themselves or others, and when non-pharmacological and primary pharmacological methods are ineffective. In these cases, special care should be taken to protect the patient from life-threatening situations (Table 4).

**Table 4:** Important recommendation points in physical restraint

• Explain the cause of restraint to the patient • Restraint belts and straps should be made of leather and be wide • Physical restraints should not use as punishment • The patient can see at least one technician • Speak with the patient in a calm and slow tone during a restraint • Take care of the patient’s head during restraint • Only restraint the patient’s hand and legs. • Check the patient’s vital signs, especially the pulse of extremities under the restraints. • Extremities should not be under pressure, • The patient should be able to move extremities under the restraints. • Do not use damaged equipment for restraining patients • The ambulance stretcher used for restraint its height should be at the lowest level • Control patient for the level of consciousness and dehydration • All actions should be documented • Do not put the patient in a prone position • Call the police • After physical restraining, pharmacological restraint should be applied	

In addition to the above-mentioned points, consider the Contraindications of restraining which include the followings [22, 23]:

- Cases in which patients used Phencyclidine (PCP) based on the family history
- Patients with recent surgery in the eye or central nervous system (Because of an increase in intra-cerebral or intraocular pressure)
- patients with a low level of consciousness or with delirium

c) Intervention in the patient family including having empathy and understanding of the critical situations and psycho-education about the conditions and places that they can attend [24].

Management of suicide emergencies requires special consideration. The expert panel suggests that suicide emergencies need a separate protocol. Moreover, while drug and alcohol poisoning and deprivation have similar symptoms with psychological emergencies, they have a completely different treatment, and they need another protocol.

## Discussion

A psychiatric emergency refers to any disturbances in a patient’s thinking, emotions, or behavior that requires immediate intervention. This disruption usually puts patients in critical conditions, which can put them, their family, and people around them in danger. These emergencies include hurting others or themselves, aggression, restlessness, acute behavioral symptoms caused by drug poisoning, depression, and severe anxiety [15].

It is essential to distinguish between the physical and psychological causes of these symptoms because it completely changes the course of treatment. In some reference books and articles, there are general protocols for managing psychotic patients [25]. But in most of the previously written protocols, the management of a symptom such as aggression or agitation has been considered [24–27]. Allen, et al. recommended different pharmacological and behavioral interventions inpatient with agitation in different situations [24, 28].

Gargia et al. suggested several recommendations on the assessment of agitation emphasize the importance of identifying any possible medical cause [29]. Most of the existing protocols are related to patient management in the hospital emergency department, and less attention has been paid to earlier stages, such as the pre-hospital stage.

In the current protocol, the first thing is the safety of technicians, their patients, and people who are in the scene. Almost all references related to acute psychotic symptoms consider the existence of a safe environment as the necessary precondition for patient management [15, 16]. These include the safety of the environment and the management of patients’ behaviors that may be harmful to themselves and others. Therefore, this protocol as well as predicting such behaviors and managing them according to international protocols is also considered.

Because of the legal aspects, police presence was also expected in the case of using mandatory treatments. This is also important for the safety of the technician, and it has been addressed in previous protocols [30]. Also, considering the priority of saving a patient’s life, early assessment of his/her vital signs is a priority [31] which we put in the primary steps of treatment. Because of the importance of diagnosing physical disorders that have symptoms similar to mental disorders, we placed making a differential diagnosis after examining vital signs.

The second point in the present protocol is to consider the overlap of many symptoms of physical illness with psychiatric disorders and to consider the differential diagnosis. Therefore, vital signs and evidence of life-threatening disorders should be examined. Impairment of the judgment and lack of co-operation in psychiatric patients can worsen the situation according to the cultural conditions in Iran.

In majority of patients’ cases, living with families and intervention of families in the process of the treatment as well as utilizing the capacity of families in the evaluation of the patient can be very helpful, which is highly considered in the present protocol. Also, in addition to the common spiritual principles associated with each patient (such as empathy, complete acceptance and being non-judgmental, etc.) paying attention to the specific religious and spiritual cultures and areas of each region that affect the therapeutic relationship with the patients is of great importance [32].

In the current protocol, like previous ones, non-pharmacological management is preferred over pharmaceutical methods. Calming the patient without medication is the priority, and drug therapy is the next priority. At the time of drug administration, in compliance with the general principles of pharmacological therapy, the priority is with the minimum dose and the oral administration root. Injectable drugs are the next priority [29]. The goal of drug therapy is to calm agitated patients without decreasing their level of consciousness [33]. However, based on available drugs and their side effects, and the possibility of drug abuse, we chose different types of drugs for the protocol. Lorazepam can be a useful drug, given the short time in the pre-hospital emergency and the need to calm the patient down with the least side effect. Lorazepam can be used in mild to moderate emergencies and in patients who are more cooperative [34].

Injectable benzodiazepines and antipsychotics such as olanzapine (considering its interaction with lorazepam and the possibility of cardiovascular collapse), ziprasidone and haloperidol are the last lines of treatment [19, 35, 36]. The use of injectable midazolam as recommended in other protocols [37] was not approved by experts despite its rapid and practical effect on sedation, because of the risk of abuse. Other antipsychotics such as aripiprazole were not approved in previous protocols like our protocol and are not recommended [29]. The use of physical restraint is proposed for patients who have not responded to primary treatments and may harm themselves and others. In some protocols, special beds with certain height are recommended for physical restraint. Given that these beds do not exist in Iran, restraint with ordinary beds and wide and leather straps was recommended. Indications, safety recommendations, and limitations for using physical restraint in our protocol are consistent with other protocols. The use of physical restraint should be accompanied by chemical restraint (use of medication to calm the patient) [22, 23]. Special attention for patients with delirium is similar to previous protocols [23].

### Study limitations

The study has several limitations. Firstly, a systematic review of the study has not been conducted and only a review of existing articles was undertaken. And secondly, Due to the limited availability of medicines in the emergency room of Iran, the suggested treatments may not necessarily be the best choices.

## Conclusion

It seems that, given the lack of similar protocols in our country in the past, this protocol can be a solution to improve emergency technician training. Such summarized protocols can be used for rapid reviews immediately before being exposed to a patient with an acute psychiatric condition.

## Supplementary information

**Additional file 1.** Pre-hospital emergency protocol for mental disorders.

## Data Availability

Not applicable.

## Abbreviations

EMS: Emergency medical service
PCP: Phencyclidine
