# Supplementary material for: First line in psychiatric emergency: pre-hospital emergency protocol for mental disorders in Iran
Source: BMC Emerg Med. 2020 Mar 16;20:19. doi: 10.1186/s12873-020-00313-2 (PMC7074981; doi:10.1186/s12873-020-00313-2)
Supplement: Supplementary file 1 — Additional file 1. Pre-hospital emergency protocol for mental disorders. [file 12873_2020_313_MOESM1_ESM.docx]

**1**

**No**

Contact Dispatch to Request Police (110)

Predict how the backup forces enter

avoidance of entering the place alone

Is it possible to enter the scene?

The scene management (security)

**Pre-hospital emergency protocol for mental disorders**

**Yes**

Overall Assessment - Requesting Help Resources

Primary assessment (Airway – Breathing - Circulation)

targeted mental and of physical history, Controlling Vital Signs (BP-O_2_sat-RR-PR-BS-GCS)

Considering the possibility of suicide

Considering the possibility of violence (Contact Dispatch to Request Police, 110)

**5**

**6**

**2**

**4**

**3**

**No**

**Yes**

**No**

**Yes**

**No**

**Yes**

Following the doctor's orders

these medications can be repeated with cardiac and blood pressure monitoring up to twice per 30 min

First line:

- Tab lorazepam 2 mg ± Tab Risperidone 2 mg **or** Tab Olanzapine 5 -10 mg **or** Tab Haloperidol 5 mg

Second-line:

- Amp Haloperidol 5 mg ± Amp Lorazepam **or** Amp Promethazine 50 mg IM

Ordered transfer to the hospital?

Following the doctor's orders

Physical restraint of the patient and the use of medications

Telephone consultation with the emergency center physician

aggressive behavior or aroused and the potential for danger to oneself or others or the possibility of escape?

Patient Support and behavioral management

Possible physical causes of symptoms

Refer to General Hospital/Medical Centers

**Coordinate with Dispatch center to transfer to the appropriate medical center**

**announce to the Destination hospital –** **Continuous Patient monitoring**

**7**

Before entering the scene, consider the following:

- Ensure the safety of the patient, technicians, and people at the scene
- pre-scene assessments of site security
- escape routes
- safe locations in the event of violence from the patient
- Assessment of risk factors for violence and predicting it
- assessment of patient’s access to weapons
- Assessment for the need for back up and the presence of police
- avoidance of entering the place alone
- using family capacities to provide security
- Patient assessment:
- Urgent physical needs by evaluating vital signs (Airway, presence of respiratory distress, and pulse)
- Obtaining mental health history including: Demographic characteristics, history of psychiatric illness, history of drug abuse, history of violence or suicide
- history of physical and primarily neurological diseases
- assessment of physical risk factors:
  - sudden onset of symptoms without previous history,
  - age younger than 12 years and older than 60 years,
  - known neurological diseases such as seizures or dementia
  - existence of neurological symptoms (ataxia, nystagmus and…)
  - complex drug regime
- Considering cultural and spiritual aspects of patients
- Assess differential diagnosis

**7**

**6**

**5**

**3**

**1**

**2**

**4**

If you decide not to transfer the patient to the hospital.

- Family psychoeducation.
- Continue contact with family by triage or by contacting hotline 123.

Important recommendation points in physical restraint

- Restraint belts and straps should be made of leather and be wide
- Explain the cause of restrain to the patient
- The patient can see at least one technician
- Take care of the patient's head during restraint
- Only restraint the patient’s hand and legs. Check the patient’s vital signs, especially the pulse of extremities under the restraints.
- Extremities should not be under pressure, and the patient should be able to move them a bit
- Do not use damaged equipment for restraining patients
- Control for the level of consciousness and dehydration
- All actions should be documented
- Do not put the patient in a prone position under any circumstances (risk of apnea)
- Call the police

Symptoms of imminent aggression include

- Motor restlessness and agitation
- The loud and threatening tone of voice
- Threatening behaviour and gestures
- Verbal Threats
- Staring and angry face mode
- Sudden behaviours (He throws the object in his hand suddenly)
- Bizarre behaviour due to delusion and hallucination
- Head trauma
- Post ictal condition
- Delirium condition
- Hypoglycaemia
- Electrolyte disturbance (Hypo natremia, hyper natremia, hypo kalemia, hyper kalemia, …)
- Hypoxia
- Encephalitis, Meningitis
- Encephalopathy (due to medical condition)
- Environmental toxicity Thyroid dysfunction
- Alcohol intoxication/ with drawal/delirium termense
- Substance intoxication/ with drawal:
- Opiums
- Amphetamins,
- kanabis
- others
- Medical intoxication/ with drawal:
- Benzodiazepines
- Anticulvansalts
- others
- Speak to the patient in a calm, measured and confident tone
- Reduce external stimuli, such as the noise and the provocative behaviour of others
- Reduce internal triggers like hunger and thirst, and offer water and food to the patient whenever possible.
- have empathic and non-judgmental attitudes and behaviours
- Appropriate acceptance of patient hallucinations and delusions
- Don't make a false promise to the patient
- Use short, simple sentences and repeat the sentences if necessary
- Listen to the patient
- Use patients’ words as much as possible
- Reassure the patient that you understand the problem
- Encourage the patient to provide information on who can help
